# Status of prevention of neural tube defects post-folic acid fortification of cereal grains in South Africa

**DOI:** 10.1017/S1368980024002271

**Published:** 2024-11-29

**Authors:** Vijaya Kancherla, Phillip Randall, Arnold L Christianson, Helen Louise Malherbe

**Affiliations:** 1 Center for Spina Bifida Prevention, Department of Epidemiology, Rollins School of Public Health, Emory University, Atlanta, Georgia, USA; 2 P Cubed, Pretoria, Gauteng, South Africa; 3 University of the Witwatersrand, Johannesburg, Gauteng, South Africa; 4 Centre for Human Metabolomics, North-West University, Potchefstroom, North-West Province, South Africa; 5 Rare Diseases South Africa NPC, Sandton, Gauteng, South Africa

**Keywords:** Neural tube defects, Folic acid fortification, Primary prevention, Cereal grains, South Africa

## Abstract

**Objective:**

Neural tube defects (NTD) are serious, life-threatening birth defects. Staple food fortification with folic acid (vitamin B_9_) is a proven, effective intervention to reduce NTD birth prevalence. Mandatory food fortification with folic acid was implemented in South Africa (SA) in 2003. This article provides an overview of NTD birth prevalence in SA, pre- and post-fortification, and evaluates current folic acid fortification regulations.

**Design:**

Fortification effectiveness data in SA were reviewed using published studies and national reports on NTD birth prevalence pre- and post-folic acid fortification. Current folic acid fortification regulations in SA were evaluated by experts.

**Setting:**

Regulations were assessed using national health guidelines, legislation and regulations. NTD birth prevalence data were sourced from the published literature.

**Participants:**

None.

**Results:**

Significant reductions in the birth prevalence of spina bifida and anencephaly and improved maternal folate levels have been achieved following the introduction of folic acid fortification in SA. However, there is poor overall regulatory compliance in some instances and a gap in current regulations that excludes the fortification of cake flour in SA.

**Conclusions:**

While the SA NTD birth prevalence has decreased by 30% post-fortification, the regulatory exclusion of cake flour fortification is a significant and growing issue. Proposed 2016 regulatory amendments to address this gap urgently require finalisation and enactment by government to prevent negating benefits achieved to date and to ensure continued improvement. Fortification monitoring requires strengthening to ensure widespread compliance with policies, particularly in underserved areas.

## Neural tube defects

Neural tube defects (NTD) are major structural birth defects, largely comprising of spina bifida and anencephaly, and are characterised by incomplete closure of the neural tube around the 28^th^ day of gestation^(1)^. Anencephaly is not compatible with life, while infants born with spina bifida have a high risk of perinatal and neonatal mortality^(1)^. Most infants that survive with spina bifida require multidisciplinary expertise, including neurosurgeons, urologists, orthopaedics, orthotics, radiologists, stoma therapists, physio- and occupational therapists and social workers^(2–6)^.

The most common cause of NTD is maternal folate insufficiency before and during early pregnancy^(7)^. Folic acid or folate is vitamin B_9_, an essential micronutrient required in early pregnancy for proper neurulation. Folate is a natural form of B_9_ available through dietary sources (e.g. lentils, beans and green leafy vegetables), while folic acid is a synthetic form of folate, available as tablets and in folic acid-fortified foods (e.g. enriched wheat and maize flour).

According to the WHO, folic acid intake is a Category 1 intervention, recommending that all women of reproductive age take 400 mcg/d folic acid (ideally before conception) to prevent NTD^(8)^. In May 2023, World Health Assembly resolution 76·19 was passed – ‘Accelerating efforts for preventing micronutrient deficiencies and their consequences, including spina bifida and other neural tube defects, through safe and effective food fortification’^(9)^. All World Health Assembly member nations were urged to invest in sustainable folic acid interventions, including food fortification, and to monitor periodically for effectiveness^(9)^.

## Birth prevalence of neural tube defect

Globally on average, there are twenty cases of NTD per every 10 000 live births^(10)^. In Sub-Saharan Africa and North-West Africa, the rate is approximately 14·2 and 17·5 per 10 000 live births, respectively^(10)^. The NTD birth prevalence varies widely globally due to different folic acid-interventions implemented, that is, supplement programmes and mandatory or voluntary food fortification policies^(11)^. In South Africa (SA), which implements mandatory fortification of wheat flour with folic acid, the latest NTD birth prevalence is estimated as 8–12 per 10 000 live births^(12,13)^.

## Neural tube defect birth prevalence in South Africa

SA is an upper-middle income country located at the southern tip of Africa with a population of just over 63·2 million people, spread over 1·2 million km^2(14)^. The NTD birth prevalence in SA was first examined in the 1990s using hospital-based studies. A study undertaken in the rural province of Limpopo reported that most infants born with an NTD died before the age of 2 years^(15)^. In 2006, Christianson *et al.* estimated the SA birth prevalence of non-syndromic NTD as 25 per 10 000 births^(16)^. Subsequently in 2019, Krzesinski *et al.* ^(13)^ reported 8–12 cases per 10 000 births. Further modelled NTD birth prevalence rates published in 2018 by Blencowe *et al.*, using 2015 births data, indicated 10 cases per 10 000 live births in SA, equating to about 1000 cases per year^(10)^. Blencowe *et al.* (2018) estimated that of the annual NTD-affected births in the SA, about 165 result in elective terminations of pregnancies, 270 result in stillbirths, and 575 result in live births^(10)^. Spina bifida alone impacts 350 live born infants per year in SA^(10)^. Many of those surviving with NTD in SA can experience premature mortality or are expected to live with lifelong health complications and associated disability^(10)^.

## Neural tube defect-related services and support in South Africa

Rates of prenatal diagnosis of NTD through ultrasonography vary provincially and between facilities. All pregnant women in SA should receive an ultrasound before 20 weeks gestation, with those screening positive for NTD (and other identifiable, priority birth defects) referred for invasive diagnostic interventions to confirm diagnosis. However, in practice, this does not routinely occur due to limited infrastructure and lack of staff trained in ultrasound screening^(17–20)^.

In SA, NTD are one of the priority birth defects notified through the Birth Defect Collection Tool, a paper-based population-based surveillance programme administered by the South African National Department of Health. The current system has been implemented since 2006, with the sole emanating publication indicating gross underreporting of all priority birth defects, including NTD^(21)^. An ongoing health system strengthening project, Ubomi Buhle (https://ubomibuhle.org.za/), is implanted at 16 primary healthcare sites across three provinces. While primarily focused on an improved understanding of the effects of medicine and vaccine exposure during pregnancy, the project also monitors and reports birth outcomes, including NTD. Ubomi Buhle is piloting an SA-adapted, surveillance version of the Global birth defects app, and if successful, implementation could be expanded elsewhere in the country^(22)^.

Pregnant women attending state antenatal care are prescribed and dispensed folic acid tablets (5 mg daily) as recommended by the Guidelines for Maternity Care in South Africa^(17)^. The South African Clinical Guidelines for Genetic Services specifically recommend folic acid supplementation as an NTD-preventative measure as part of pre-conception and pre-pregnancy care to prevent birth defects and to use staple foods fortified with folic acid^(18)^. While these and other relevant guidelines exist^(17,18,23)^, their practical implementation remains limited, fragmented and under-resourced.

Health education and awareness of NTD is an essential part of family planning and ante-natal care, highlighting the importance of folic acid supplementation and use of folic acid fortified staple foods, throughout the life cycle and particularly preconception. This is particularly important when planning subsequent children following the birth of an NTD-affected child. Education and psychosocial support for affected families is offered by the Association for Spina Bifida and Hydrocephalus South Africa, founded in 2014. Association for Spina Bifida and Hydrocephalus South Africa (www.asbah-sa.org/) aims to protect and promote the interests of those impacted; support measures to prevent or reduce NTD and advocate for accessible and equitable medical, educational, vocational, social, and rehabilitation services.

## Folic acid food fortification in South Africa

South Africa first implemented a nation-wide food fortification programme in October 2003, after rural black women of childbearing age were found to have insufficient folic acid intake^(24,25)^. The fortification regulation required the milling industry to add specific formulations of vitamins and minerals, including folic acid, to certain wheat flours and maize meals. Regulations were subsequently amended in 2008^(26)^ and are currently implemented as detailed in Table 1. The associated cost of fortification and the amount passed onto the consumer were found to be nominal, translating to only a ZAR0·01 (one cent) increase per loaf of bread and ZAR0·02 per kilogram of maize meal^(27)^.

**Table 1:** Current (2008) South African regulations relating to folic acid fortification^(26)^

Foodstuff	Definition and fortification	Exclusions
Wheat Flour	1·4 mg of folic acid per 1 kg. All milled, dry and uncooked wheat products with an ash content of >0·60 % (moisture free).	Crushed wheat, pearled wheat, semolina, self-rising flour and all flours (including wheat flour) with an ash content of <0·60 % (moisture free). **Cake flour** (ash content <0·60 %) contributing 38 % of the market, commonly used in domestic environments and small bakeries, **is not fortified.** Approximately 58 % of wheat flour (bread and brown flours) is fortified.
Maize Meal/Flour	2 mg of folic acid per 1 kg. All milled, uncooked maize products, including super, special, sifted and unsifted maize meal.	Samp, grits, maize rice and maize flour (particle size makes fortification problematic).
Wheat Bread	All baked bread prepared with/containing ≥90 % fortified wheat flour (moisture free).	Excludes ‘bad foods’, e.g. sweet biscuits, confectionary, etc. Aimed to capture the bread market as people eat products, not flour.

Currently, maize flour fortification occurs in both domestically produced and imported products, including flour that is used in the household and processed foods. The fortification concentration is 2 mg of folic acid per 1 kilogram of maize flour^(28)^. Wheat (bread) flour is also fortified, including domestically produced and imported products, used in household and processed foods. The concentration of folic acid added to wheat flour is 1·4 mg per 1 kilogram of flour^(28)^. Current regulations exclude the fortification of cake flour^(26)^. While this exclusion of cake flour is addressed in additional regulatory amendments proposed in 2016^(29)^, 8 years later these remain unfinalised, despite cake flours accounting for almost 30 % of wheaten products manufactured in SA^(30)^.

## Effectiveness of folic acid fortification in South Africa

In 2007, Modjadji *et al.* ^(31)^ used serum and red blood cell folate levels among non-pregnant women of childbearing age in Dikgale Health and Demographic Surveillance System in a rural district of Limpopo Province, SA. These blood markers are commonly used biomarkers to study the effectiveness of folic acid fortification. In the study, median serum folate concentration increased from 3·58 to 10·51 ng/ml following fortification, while the median red blood cell (RBC) folate concentration rose from 227·01 to 429·29 ng/ml. The proportion of women with low serum folate levels (<3 ng/ml) decreased from 16·3 % to 0 % post-fortification, while those with low RBC folate (<164 ng/ml) decreased from 26·4 % to 1·9 %^(31)^. This study showed the benefits of folate fortification in improving serum and RBC folate concentrations in SA target populations.

In 2008, Sayed *et al.* ^(32)^ compared the birth prevalence of NTD pre-folic acid fortification to post-fortification using data from twelve hospitals in SA. The study showed a 11 % reduction in anencephaly and a 42 % reduction in spina bifida birth prevalence. The perinatal mortality rate associated with NTD also declined by 65·9 % in the post-fortification period, with the total national infant mortality rate declining by 38·8 %. The cost–benefit ratio of food fortification was estimated to be 46:1, showing the great benefit of this intervention in SA^(32)^.

## Status of food fortification monitoring for adequacy of folic acid

While previous studies demonstrate the effectiveness of folic acid fortification in SA, continuous monitoring is needed to ensure implementation and full compliance as per national policy. To encourage this, WHO has published a guidance manual for millers, regulators and for program managers monitoring fortification^(8)^. These regulations require suppliers of micronutrient premix to register with the Department of Health and to submit analysis reports on their premixes at 6-monthly intervals undertaken by an independent laboratory accredited with the South African National Accreditation System^(26,33)^.

Additionally, Food Control Sections within both National and Provincial Department of Health have trained ±2000 inspectors to conduct onsite checks at mills countrywide. While the original regulations^(24)^ specified that samples should be taken at the ‘point of manufacturing, importation or sale’, this was later amended to exclude the point of sale for legal reasons related to traceability, counterfeiting, etc^(26)^.

While the South African government intended premix suppliers to provide Department of Health with data related to sales volumes (kg) of each premix type, this met with resistance from suppliers due to confidentiality and competition issues. Information relating to the internal stability of micronutrients is required from premix suppliers with annual registration, although compliance levels are not publicly available.

Whilst SA does not publish fortification data, it is believed that compliance by the large millers, who dominate circa 75 % of the maize market and almost all the wheat market, is now at 80 %^(28)^. This is marred by the exclusion of cake flour that remains unfortified.

## Conclusion

The birth prevalence of NTD in SA decreased by 30% after folic acid fortification was introduced in 2003^(32)^. Further reductions are possible through improved, comprehensive, effective monitoring activities, and the finalisation and enactment of regulatory amendments proposed in 2016 to address the current exclusion of folic acid fortification of cake flour^(29)^. The World Health Assembly 2023 Resolution 76·19 urges member nations to monitor the quality of fortification, along with periodically measuring folate insufficiency in women of reproductive age^(9)^. Resolution 76·19 also recommends the development and implementation of financing mechanisms to effectively implement fortification and support monitoring activities for compliance with the policy. These collective actions require urgent implementation to ensure greater accountability and improved health outcomes.
